# Developmental changes in brain activation during novel grammar learning in 8-25-year-olds

**DOI:** 10.1016/j.dcn.2024.101347

**Published:** 2024-01-20

**Authors:** W.M. Menks, C. Ekerdt, K. Lemhöfer, E. Kidd, G. Fernández, J.M. McQueen, G. Janzen

**Affiliations:** a Donders Institute for Brain, Cognition and Behaviour, Radboud University and Radboud University Medical Center, Nijmegen, the Netherlands; b Behavioural Science Institute, Radboud University, Nijmegen, the Netherlands; c Max Planck Institute for Psycholinguistics, Nijmegen, the Netherlands; d ARC Centre of Excellence for the Dynamics of Language, Australian National University, Canberra, Australia; e School of Literature, Languages, and Linguistics, Australian National University, Canberra, Australia

**Keywords:** Grammar learning, Development, Functional magnetic resonance imaging (fMRI), Second language acquisition, Initial learning

## Abstract

While it is well established that grammar learning success varies with age, the cause of this developmental change is largely unknown. This study examined functional MRI activation across a broad developmental sample of 165 Dutch-speaking individuals (8–25 years) as they were implicitly learning a new grammatical system. This approach allowed us to assess the direct effects of age on grammar learning ability while exploring its neural correlates. In contrast to the alleged advantage of children language learners over adults, we found that adults outperformed children. Moreover, our behavioral data showed a sharp discontinuity in the relationship between age and grammar learning performance: there was a strong positive linear correlation between 8 and 15.4 years of age, after which age had no further effect. Neurally, our data indicate two important findings: (i) during grammar learning, adults and children activate similar brain regions, suggesting continuity in the neural networks that support initial grammar learning; and (ii) activation level is age-dependent, with children showing less activation than older participants. We suggest that these age-dependent processes may constrain developmental effects in grammar learning. The present study provides new insights into the neural basis of age-related differences in grammar learning in second language acquisition.

## 1 Introduction

The acquisition of formal components of language, such as phonology and grammar, is generally more successful if an individual learns a target language in childhood (DeKeyser, 2013, Hartshorne et al., 2018, Johnson and Newport, 1989, Long, 1990, Newport, 1990, Weber-Fox and Neville, 1996). With respect to grammar, our focus here, past research has suggested that the ability to attain native-like proficiency declines some time between childhood and late adolescence. Although the exact timing of the effect is debated, it is uncontroversial that grammar learning success is moderated by age. The neural mechanisms responsible for this developmental shift, however, are largely unknown. In the current paper, we report on a large-scale functional Magnetic Resonance Imaging (fMRI) study that investigated the neural structures that support the initial phase of grammar learning of a novel language in individuals aged 8–25 years. Accordingly, we provide the largest and most comprehensive fMRI investigation of second language (L2) initial grammar learning to date.

Grammar learning is a complex process that relies on multiple neural networks, one of which is the language network. Within this left-dominant fronto-temporal network, researchers have identified several regions that specifically support grammatical processing. Particularly, the triangular and opercular parts of the inferior frontal gyrus (IFG) have most often been linked to grammatical processing and are thought to be an important language hub within the brain (Fedorenko and Blank, 2020). Other cortical regions that are often activated during grammar-related tasks are the bilateral anterior insula, the medial frontal gyrus, and the bilateral angular gyrus (Matchin and Hickok, 2020, Tagarelli et al., 2019). Besides the language domain-specific network, multiple other neural networks that subserve broad domain-general functions (e.g., memory, attention, and cognitive control) seem to contribute to grammatical processing (Campbell and Tyler, 2018, Deldar et al., 2020). Interestingly, most of these aforementioned language-related brain regions have also often been linked with working memory processes such as maintenance, encoding, and retrieval (Deldar et al., 2020, Owen et al., 2005, Rottschy et al., 2012). For example, some authors suggest that the left inferior frontal gyrus is not language-specific but is associated with other domain-general cognitive processes such as working memory maintenance, which in turn support language processing (Fiebach et al., 2005, Hagoort, 2013).

It is plausible that the maturation of both the language-specific and domain-general networks in the brain could be the reason that grammar learning changes significantly with age. Neurodevelopmental studies have shown that brain structures develop rapidly in early childhood, followed by continuous gradual maturation during puberty and early adulthood (Fuhrmann et al., 2022, Gogtay et al., 2004). Besides structural changes, altered functional activations have been linked to brain maturation. One example is the shift from a diffuse to a more focal brain activation pattern in relation to age during both language-related and non-linguistic cognitive control tasks (Brauer and Friederici, 2007, Durston et al., 2006). This shift in activation might be a consequence of the increased functional specialization and reorganization of the maturing brain during development. Interestingly, the language network, and parts of the domain-general networks, are predominantly located within the (pre)frontal and temporal regions of the brain. These brain regions are notable because they follow a long maturation trajectory in comparison to other parts of the brain, not becoming fully mature until around 20 years of age (Tamnes et al., 2017). Consequently, the maturation trajectories of the language network, and partly the domain-general networks, are highly plausible as the neural source of age-related constraints on grammar learning ability.

Due to the limited number of developmental studies, little is known about the developmental trajectory of the language network underlying grammar learning during childhood and adolescence and its relation with grammar learning ability. The handful of studies that have investigated this topic in children and/or teens have used extreme age-group designs and therefore cannot be used to make detailed inferences about developmental change (Sakai, 2005, Tatsuno and Sakai, 2005). Nevertheless, these studies have provided important evidence that age-related differences in grammatical processing can be observed in neural structures (Sakai, 2005, Tatsuno and Sakai, 2005). For example, activation within the left IFG is increased for teenagers compared to young adults during grammatical processing (Tatsuno and Sakai, 2005). Although these results are promising, a study that examines a continuous age range is required in order to determine whether the behavioral observation of age-related changes in grammar learning ability can be linked to a developmental neural shift.

In comparison to the dearth of studies investigating the neural underpinnings of new grammar learning in development, more is known about the development of domain-general networks, especially for memory-related processes. Behaviorally, researchers have found that both declarative and working memory abilities increase linearly from early childhood through adolescence (Finn et al., 2016, Gathercole et al., 2004, Hartshorne and Germine, 2015, Maylor and Logie, 2010, Simmonds et al., 2017). Conversely, numerous studies observed that procedural (e.g., implicit) learning seems unaffected by age and appears already adult-like in late childhood, although sometimes pre-teen children perform worse than adults (Finn et al., 2016, Gathercole et al., 2004, Janacsek et al., 2012, Simmonds et al., 2017, Thomas et al., 2004, Zwart et al., 2019). Age effects on memory performance are associated with structural and functional differences in the brain (Bathelt et al., 2018, Thomas et al., 2004, Thomason et al., 2009). For example, even though children and adults recruited similar regions during working memory tasks, adults exhibited more activation than children (Thomason et al., 2009). Additionally, several studies have identified developmental shifts (e.g., from subcortical to more cortical regions) in the neural substrates associated with improved working memory and procedural memory (Bathelt et al., 2018, Simmonds et al., 2017, Thomas et al., 2004, Thomason et al., 2009). Thus, ample evidence indicates that working memory, in particular, improves during adolescence, and that the maturation of brain regions necessary for memory-demanding operations could be an important driving factor.

From the viewpoint of neural development, language learning thus represents a developmental paradox: whereas adults have fully matured memory and language networks that support rapid learning across many domains, children are ultimately better language learners, in spite of their comparatively limited cognitive resources and still-developing brain networks specializing for higher cognitive processes. To date, most research bearing upon this issue has been retrospective and behavioral in nature. To better unravel this paradox, we need to examine how all brain areas and neural networks that support grammatical processing are implicated in learning as they mature across the developmental window in which the potential for native-like language learning closes.

In the current study, we report a large-scale cross-sectional fMRI study that aimed to determine the neural underpinnings of age-related changes during initial grammar learning. Our study was developmental, testing participants between the ages of 8 and 25 years on their ability to learn, implicitly, a new grammatical system based on Icelandic morphosyntax. We developed an adaptive grammaticality judgment task that allowed assessment of individual performance on a sequentially introduced set of grammar rules. This task thus enabled us to investigate individual learning for all ages and avoid ceiling or floor effects. We asked two main research questions: 1) How does age affect grammar learning performance in the early phase of learning new grammar? 2) What functional brain regions correlate with the hypothesized age-related variability in grammar learning ability? Given the delayed maturation of the brain, we hypothesized that activation in both the language network and memory-related regions would correlate with age-related grammar learning performance. With our broad dataset, we were able to test several hypotheses. One possibility is that children rely on a qualitatively different network of brain areas than adults, and during development a gradual or abrupt shift occurs from a child-like to a more adult-like brain network (Ullman, 2004). Another possibility is that children and adults use a similar network of brain areas, but that its activation is age-dependent.

## 2 Methods

### 2.1 Participants

We recruited 195 right-handed participants aged 8 - 25 years. All were raised monolingually, with Dutch as their native language (L1). Adults were recruited through social media, posters, and a specialized research participation system at Radboud University. Children and teenagers were contacted through flyers and posters at their schools, libraries, and community events. Individuals without any MR contraindication, history of neurological or psychiatric treatment/illness, a current psychiatric diagnosis, language impairments or learning disabilities were included. Thirty participants were excluded due to equipment malfunction (N = 6), incomplete data sets (N = 10), poor fMRI data quality (N = 11), reading difficulties (N = 2), and a late autism diagnosis (N = 1). As a result, we included a total of 165 participants (M = 17.88, range = 8.26 - 25.97 years) in the analyses. None of the participants had learned our training language, Icelandic, before this experiment, although many had experience in the most common second languages learned in the Netherlands (i.e., English (100%), German (65%), French (70%), and Spanish (15%)). All participants gave written informed consent; caregivers gave additional written informed consent for underaged participants. The study was approved by the regional ethics committee CMO Arnhem-Nijmegen (2018–4561; 2014–288).

### 2.2 Procedure

Participants performed a grammaticality judgment task (GJT) in two MRI sessions, once before and once after 5 days of grammar training at home. The Icelandic words used throughout this experiment were cognates in Dutch (for the full word list see Menks et al., 2022). Training participants on Icelandic-Dutch cognates increased the likelihood that our results reflected grammatical learning independent of lexical learning.

#### 2.2.1 Familiarization and home training

**Fig. 1:**
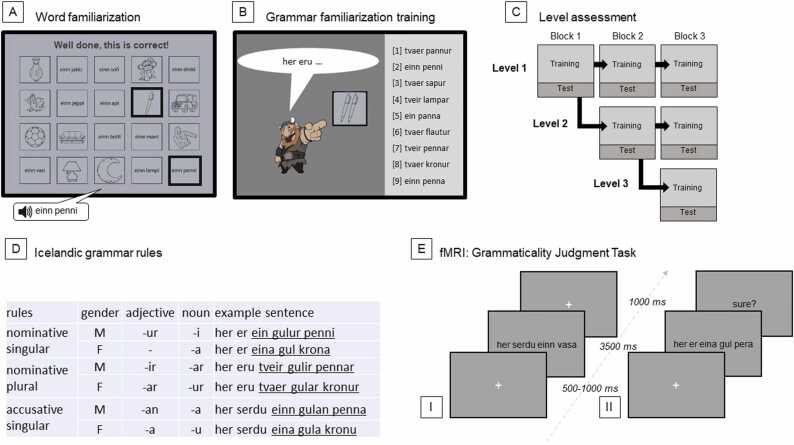
Overview and examples of the [A] word and [B] grammar familiarization tasks, as well as [C] the level assessment during the familiarization phase. [D] Examples of the Iceland grammar rules. [E] The fMRI [I] GJT (before the 5-day home training) and [II] GJT (after the training). (For further information, see Menks et al., 2022).

First, participants learned the Icelandic words needed for the first MRI session through a word familiarization task. Participants were trained twice, on separate days, through a repeating word-picture memory game until they reached a perfect score (i.e., answering all items correctly); for an example see Fig. 1A.

Second, before the first MRI session, participants completed a matching game of Icelandic sentences and images during which they were implicitly familiarized with a subset of Icelandic morphosyntactic rules: inflection of word phrases based on gender (masculine/feminine), number (singular/plural), and case (nominative/accusative; for more details see Fig. 1). Importantly, the Icelandic rules were not explained during any part of the experiment, instead, the correct answer was shown after each trial, irrespective of whether the answer given was correct or incorrect. Since we included adults as well as young children, our participants differed in grammar learning capacity. Therefore, the task was adaptive: during the familiarization phase, participants could reach three levels of grammar difficulty to avoid ceiling or floor effects in learning performance across ages (see Fig. 1C). For all levels, participants learned the morphosyntactic rules for the Icelandic noun phrases; in level 2 and level 3 participants additionally learned to inflect adjectives for masculine nouns and all nouns, respectively. Between the two MRI sessions, participants completed five days of home training. During each training day, participants played a similar grammar training game as in the familiarization phase, except that participants remained at the level they reached in the initial familiarization phase.

#### 2.2.2 fMRI task

During each MRI session, participants performed the GJT in which they judged whether the test sentences were grammatically correct or incorrect. The task consisted of 192 trials divided over a baseline (25%) and a grammar condition (75%). During the baseline (B) participants saw two identical Icelandic or scrambled words, both of which had a similar length to the Icelandic phrases tested in the grammar condition. The participants were asked to indicate whether they had learned these words. The grammar condition consisted of three types of Icelandic sentences (i.e., a carrier phrase *her eru* ‘here is’ followed by the target noun phrase) that either followed (67%) or violated (33%) the implicitly learned grammar rules. In the first MRI session, each GJT trial consisted of a white fixation cross, followed by an Icelandic sentence in black letters on a light-gray screen for 3500 ms and ending with another white fixation cross for 1000 ms, see Fig. 1E I). In the second MRI session, the GJT trials were identical to the first session, except that the participants had to indicate if they were certain of their response at the end of each trial, replacing the last 1000 ms fixation cross of the trial (see Fig. 1E II).

### 2.3 MRI acquisition

Functional MRI data were acquired on a 3 T MAGNETOM Skyra (Siemens AG, Germany) MR scanner using a 32-channel head coil. For functional images, we used a multi-echo multiband T2 * -weighted EPI sequence with the following parameters: repetition time (TR) = 1500 ms; echo time 1 (TE1) = 12.4 ms, TE2 = 34.3 ms, TE3 = 56.2 ms; slices = 51, interleaved slice order; slice thickness = 2.5 mm; multiband acceleration factor = 3; FOV = 210 × 210×128 mm; flip angle = 75°; voxel size = 2.5 × 2.5 × 2.5 mm. Slices were angulated in an oblique axial manner to reach whole-brain coverage (except for a part of the cerebellum). Subsequently, fieldmap data was recorded for distortion correction and consisted of one phase difference image and two magnitude images at two different echo times: TR = 576 ms; TE1 = 4.3 ms, TE2 = 6.79 ms; slices = 51, interleaved slice order; slice thickness = 2.5 mm; bandwidth = 804 Hz/Px; FOV = 210 × 210×128 mm; voxel size = 2.5 × 2.5 × 2.5 mm. Additionally, at the first and second MRI session a T1-weighted anatomical scan was acquired. At the last session a T1 image was collected using a Magnetization Prepared Rapid Gradient Echo (MPRAGE) sequence: TR = 2000 ms; TE = 2.01 ms; matrix size = 256 × 256; field-of-view (FOV) = 256 mm; flip angle = 8°; voxel size = 1 mm; slice thickness = 1 mm, 192 sagittal slices covering the entire brain. Parallel imaging (iPAT = 2) was used to accelerate the acquisition, resulting in an acquisition time of 4 min and 40 s. At the first session a MP2RAGE (i.e., a modified MPRAGE) sequence was implemented with the following parameters: TR/TI1/TI2 = 5000/700/2500 ms; matrix size = 256 × 216; FOV = 256 mm; flip angle1 = 4°; flip angle2 = 5°; voxel size = 1 mm; slice thickness = 1 mm, 224 sagittal slices covering the entire brain. Parallel imaging (iPAT = 4.6) was used to accelerate the acquisition time to a duration of 4 min.

### 2.4 Data analyses

#### 2.4.1 Behavioral analyses

Performance on the GJT was analyzed using RStudio (R-4.1.3). For the behavioral analyses, we used a mean score of the pre- and post-training MRI session as a measure for overall grammar learning success. All participants scored above chance level (50%) in our task. To be able to compare scores between the grammatical difficulty levels, we adjusted each participant’s performance score based on their reached level, i.e., level 1 (50%), level 2 (75%), and level 3 (100%). In this way we avoided ceiling and floor effects but are also able to compare all participants independently on their level. Based on the existing developmental literature, we expected that age effects on grammar learning were likely to follow a quadratic or cubic function instead of a simple linear function. For this reason, we investigated age effects by fitting all three functions using a regression model. Additionally, we applied a LOESS—a local polynomial regression-smoothing procedure—curve fitting function to visually assess the model-free relationship between age and grammar learning performance (degree =2, span =0.75, method="loess", parametric=FALSE, drop.square = FALSE, normalize=TRUE). This approach is qualitative and useful for identifying data patterns that may be overlooked with curve-fitting procedures that assume an a-priori shape (Cleveland and Devlin, 1988).

#### 2.4.2 fMRI preprocessing

Functional MRI data was preprocessed using fMRIprep (v22.0.1), an open-source software suite designed to increase reproducibility and develop common best practices for image processing (see supplement for more details). Subsequently, the data were analyzed statistically using a general linear model (GLM) and statistical parametric mapping using SPM12. To compare brain activation for grammatical processing versus baseline, we included three explanatory variables in the first model for each session: grammar-correct trials, baseline-correct trials, and trials-of-no-interest comprising all incorrect and no response trials. We included all participants that had a minimum of 50% of correct grammar trials. Furthermore, we checked that age-related differences in the number of correct grammar trials had no effect on our final results by reanalyzing the data using only the first 40% of all correct grammar trials per participant. Additionally, we added 24 regressors of no interest comprising six motion regressors (translation and rotation in x, y, and z axis), and 18 derivatives of these motion regressors into the design matrix. A high pass filter was implemented using a standard cut-off of 128 s to remove low frequency effects from the time series. For statistical analysis, contrast parameter images for (grammar > baseline) were generated for each participant per session and then subjected to a second-level analysis.

#### 2.4.3 fMRI data analyses

At the group level, data analysis proceeded in four steps:

1) In the first analysis, we examined patterns of activation associated with the GJT collapsed across age: the grammar vs. baseline contrast allowed us to document that we observed the well-established patterns of activation evoked in the GJT.

2) The next analyses addressed which brain regions are linked to the age-related difference in GJT scores, independent of individual differences. For this, we ran a second-level regression analysis with the extracted LOESS function as the continuous variable, allowing us to assess the main effect of the model-free LOESS function during the GJT.

3) The third step of the analysis explored follow-up post-hoc comparisons between two age-groups as defined by the analyses in (2), before and after the behavioral turning point. To accomplish this, we transformed age from a continuous variable into a categorical factor with two age levels: a young group (ages 8 till 15.4) and an older group (ages 15.4–25.9).

4) Finally, to examine individual differences in grammar learning ability in relation to neural activation patterns that are independent of age, we applied a multiple regression analysis for each group separately using GJT scores as predictor while regressing out age.

## 3 Results

### 3.1 Behavioral results: grammaticality judgment task

**Fig. 2:**
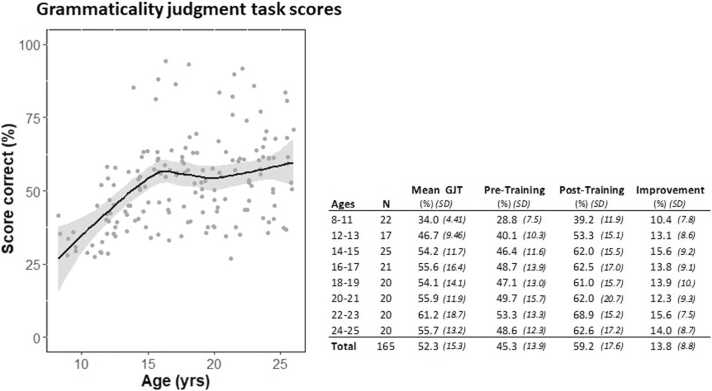
An overview of the grammaticality judgment task (GJT) scores. [Left] Mean GJT (average of pre- and post-training) scores are plotted against age overlaid with a fitted line by a LOESS function (black). The shaded areas indicate the confidence interval (95%). [Right] An overview of the mean, pre-training, post-training, and improvement scores on the GJT of the sample by age group.

We assessed the average accuracy in the GJT for each participant combined over the two MRI sessions, i.e., before and after the grammar training. The table in Fig. 2 shows the mean GJT scores, as well as the scores for each separate MRI session and the improvement after training for each age group. Overall GJT performance increased non-linearly with age: both a quadratic (*R*^*2*^ =.20, *F*(2, 162) = 20.7, *p* < .001) and a cubic (*R*^*2*^ =.21, *F*(3, 161) = 14.2, *p* < .001) curve equally better fitted the data compared to a linear function (*R*^*2*^ =.16, *F*(1, 164) = 30.5, *p* < .001). These nonlinear, parametric curves are based on a-priori assumptions which makes them unsuitable to assess any sharp changes, such as turning points, in the data. Therefore, we applied a local polynomial regression (LOESS) function to investigate the model-free relationship between age and grammar learning performance (*R*^2^ =.23). Within this dataset, the LOESS curve follows a steep increasing linear line from the age of 8 until the age of 15 to 16, where the slope becomes zero (see Fig. 2). After this point, the curve was less affected by age, showing a brief decline followed by a slight increase. To determine the exact breakpoint of the curve, we performed a break-point analysis using Bayesian inference with the RSTAN package of R to fit our model (Stan Development Team, 2019). The mean estimate of the breakpoint was determined at 15.4 years, with a confidence interval (C.I.) of 13.0–18.0 years. The estimated slope before the breakpoint is about 0.04 with a 95% C.I. from 0.02 to 0.06, whereas after the breakpoint the slope is close to 0.00 (C.I. −0.01 to 0.01). To investigate whether age is still a predictor of GJT performance after the age of 15.4, we split the group—based on this breakpoint analysis—into a young (8 till 15.4 years) and an older (15.4–25 years) group. A regression analysis carried out separately for the two groups showed a strong linear age effect (β = 0.03) on grammar learning performance in the young group (*R*^2^ =.28, *F*(1,52) = 20.49, *p* < .001), whereas age did not explain the grammar learning variation in the older group (*R*^2^ =.00, *F*(1,109) = 0.03, *p* = .860). Thus, there is an age-related discontinuity in the behavioral data.

### 3.2 Neuroimaging results

#### 3.2.1 Overall group activation during the grammaticality judgment task

Whole-group fMRI analyses identified large clusters of brain activation during the GJT, for the contrast of grammatical sentences versus baseline (see Fig. S1 and Table S1). For both the pre- and post-training session, activation increased in brain regions that are known to be involved in both grammar and memory processing, such as the superior and inferior part of the parietal lobe and superior and middle frontal regions encompassing the anterior insula and IFG. Additionally, activation was observed in the occipital lobe, cerebellum, and several subcortical structures such as the hippocampi and caudate nuclei.

#### 3.2.2 Brain regions associated with the age effect in GJT performance

**Fig. 3:**
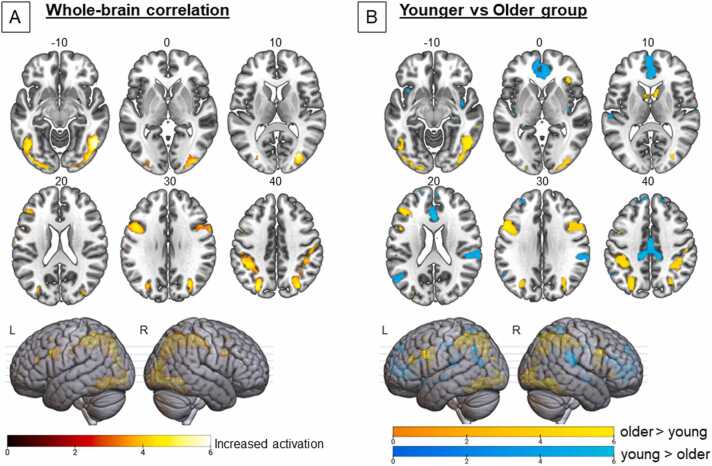
Statistical parametric maps (cluster-level, *p* < .05 FWE-corrected) displaying [A] whole-brain increased (red-yellow) activation during grammatical processing in relation to the LOESS fitted curve (i.e., the estimated combined effect of age and grammar learning performance) during the pre-training session, and [B] between-group activation comparison of the older group (15.4–25 years) to the young group (8 until 15.4 years) displaying whole-brain increased activation for the older group (yellow) and increased activation for the young group (blue) during grammatical processing in the pre-training session (MNI space).

Our behavioral analyses revealed a strong positive correlation between age and grammar learning performance, especially between the age of 8 and 15.4 years. To focus on the age-related differences and not on the individual differences within the behavioral data, we extracted the LOESS fitted curve (i.e., predicted grammar performance based on age). In other words, we calculated for each participant the predicted GJT value based on their age on the LOESS fitted curve. These values were used as a predictor for brain activation within our whole-brain regression analyses. For both the pre- and post-training session, this analysis revealed significant associations between predicted grammar score and activation in brain regions such as the bilateral IFG, fusiform gyrus, right inferior temporal gyrus, and the bilateral inferior parietal gyri (see Fig. 3A, Fig. S2A and Table S2). Direct comparison between the pre-training and the post-training session revealed modest training effects in relation to the age-related differences in grammar performance (see Fig. S2B). Before training, age-related learning performance was linked with activated clusters within the left precentral gyrus as well as the right inferior/middle temporal gyrus. After training, the age-related learning performance was associated with more activation within the left middle temporal gyrus. As part of a larger study, we have collected data about the participants’ grammar proficiency in their native language (L1; Dutch). Additional analyses indicated that the L1 grammar proficiency did not influence the age-related activation patterns observed (see Fig. S4).

#### 3.2.3 Group comparison before and after the turning point

To decompose the significant relationship between age and grammar learning performance, we split the sample into two groups (i.e., young and older) based on the discontinuity observed in the behavioral data. We then conducted a group comparison to further explore age differences in grammatical processing before and after this turning point (15.4 years). For both sessions, the older group showed a larger set of activated brain areas compared to the younger group that encompassed the bilateral inferior parietal gyrus, bilateral IFG, bilateral caudate nucleus, and anterior part of the right insula (see Fig. 3B and Table S3). In contrast, the younger group had more activation within the bilateral superior temporal gyri, posterior part of the right insula and the anterior/middle part of the cingulate cortex. After the home training, both groups exhibited similar brain activation patterns as during the pre-training (see Fig. S3B); however, small training effects were visible within the post/precentral regions, where the older group showed more deactivation after the home training in comparison to the younger group.

#### 3.2.4 Neural correlates of grammar learning performance independent of age

**Fig. 4:**
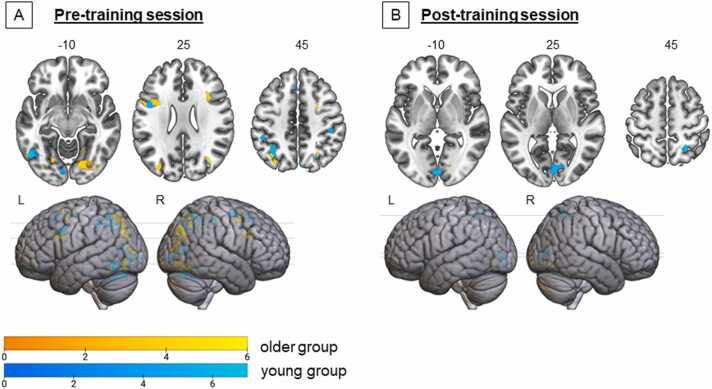
Statistical parametric maps (cluster-level, *p* < .05 FWE-corrected) for the young group (8 until 15.4 years; blue) and adult group (15.4–25 years; yellow) displaying increased activation in relation to grammar learning performance, independent of age, during the GJT at the [A] pre-training session and [B] post-training session.

Additional whole-brain regression analyses were performed where grammar learning performance was added as a predictor whilst controlling for age (Fig. 4) separately for the two age groups. Activation in several brain regions was predictive of overall grammar learning performance, especially in the young group, but only before the home training: the left IFG, left inferior temporal gyrus, bilateral inferior/superior parietal gyrus, and right middle frontal gyrus. After training, only activation within the right superior parietal gyrus, and bilateral calcarine gyri remained predictive of grammar learning. In the older group, GJT performance before training was correlated with increased activation in the left superior parietal gyrus, cerebellum, left fusiform gyrus, left IFG, bilateral middle frontal gyrus, and right middle occipital gyrus/angular gyrus for the pre-session. After training, only one cluster remained a significant predictor, the right lingual gyrus.

## 4 Discussion

In this study, we investigated the neural correlates underlying age-related variability in grammar learning. Unlike most studies that have investigated age-related effects indirectly, we tested a large developmental sample during the first stages of acquiring a new grammatical system, allowing us to uncouple age from linguistic experience. We identified a clear turning point in age-related effects on grammar learning during late adolescence, after which age had no effect on learning. Importantly, we also took a significant step towards understanding the neural correlates underlying this developmental shift. Our results demonstrate that activation patterns within the working memory and grammar-related areas are predictive of age-related grammar learning performance.

Behaviorally, we observed adults outperform children, which runs counter to the observation that children *ultimately* reach higher proficiency in a new language (at least when immersed in it; Hartshorne et al., 2018, Johnson and Newport, 1989). On the other hand, this finding is in line with previous studies demonstrating that adults and adolescents acquired a second language faster than children in the early learning phase (Ferman and Karni, 2010, Hashizume et al., 2014, McNealy et al., 2011, Takashima et al., 2019, Tatsuno and Sakai, 2005). Adding to this paradox is our finding of an abrupt discontinuity in the performance-age function at age 15.4 years, which resembles Hartshorne et al.’s (2018) deduced turning point in learning ability at age 17.4. Note, however, that the latter is a point at which learning rate (as a predictor of changes in L2 proficiency with age) suddenly drops, while at our turning point, learning performance reaches a plateau. To take a first step to unravel this developmental paradox, we examined the underlying neural correlates of initial grammar learning processes.

We investigated where specific patterns of brain activation could explain our observed age-related change in grammar learning. Indeed, activation within several cortical regions located in frontal, parietal, and the temporal parts of the brain were linked with the age-related differences in grammar learning. The home training had no noteworthy effect on the activation patterns. These activation patterns overlap largely with the working memory (fronto-parietal) network and partly with the left-dominant syntax network (Emch et al., 2019, Kaan and Swaab, 2002, Tagarelli et al., 2019). For instance, activated areas such as the bilateral inferior parietal gyri, angular gyri, and inferior frontal gyri are generally linked with domain-general working memory processes (Borst and Anderson, 2013, Emch et al., 2019, Owen et al., 2005, Rottschy et al., 2012). The left IFG, however, is also known as a domain-specific language region that is responsible for grammatical integration (Deldar et al., 2020, Tagarelli et al., 2019, Tao et al., 2021). Moreover, some researchers have proposed that the left inferior frontal cortex is specifically involved in syntactic working memory processes, where both language specific and domain-general regions lie side by side in the left IFG (Fedorenko et al., 2012, Fiebach et al., 2005). Although the contribution of the IFG in grammar processes is still under debate, working memory has frequently been linked with increased second language performance in both adults and children (Fiebach et al., 2005, Linck et al., 2014, Martin and Ellis, 2012, Santi and Grodzinsky, 2007, Verhagen and Leseman, 2016).

We also compared the overall brain patterns of the participants in groups falling before and after the behavioral turning point in age (15.4 years). As expected, older learners exhibited more activation within the working memory and syntax related areas. In contrast, younger participants recruited more middle and superior temporal regions as well as the right posterior insula in comparison to the older group. The older group recruited the working memory network more, yet activation was not highly predictive of their grammar learning performance. This indicates that after the turning point, other behavioral or neural factors, or a combination of such factors, are likely to be better predictors for the observed individual differences in grammar learning. Although the working memory and syntax related areas were overall less activated in younger participants, activation within those areas increased in relation to grammar learning performance. Taken together, these results suggest that children, adolescents, and young adults recruit largely similar brain networks to acquire a new grammar. We thus have evidence against the simple hypothesis that age-related changes in grammar learning ability arise because children rely on a qualitatively different network of brain areas than adults do. Instead, it appears that children and young adolescents use the same networks but do not recruit them to the same degree as young adults, since activation continues to increase with age into late adolescence. Additionally, the data suggest that the better the young participants perform during our GJT, the more these networks are recruited in an adult-like manner.

Our findings therefore suggest that the maturation of the working memory and syntax related areas could underlie the observed age-related variability in grammar learning. Indeed, behavioral studies have demonstrated that working memory capacity increases linearly from age 4 to late adolescence, after which working memory performance is adult-like and remains stable until old age (Gathercole et al., 2004, Simmonds et al., 2017). The developmental trajectory of working memory performance largely overlaps with the age effects observed in our study, which similarly asymptotes around late adolescence. Furthermore, the activation patterns underlying these age-related effects are located within brain regions where development is ongoing during adolescence, which in turn influence general memory and language processes (Bitan et al., 2007, Ofen et al., 2012, Tamnes et al., 2017). Brain maturation could therefore be the driving factor for the age-related changes in grammar learning performance observed in this study. This age-related increase in brain activation is in agreement with two meta-analyses that observed similar increased brain activation in relation to age for semantic processing (Enge et al., 2021, Weiss-Croft and Baldeweg, 2015).

Even though younger learners have a slow start in grammar learning, they have a higher chance to ultimately reach native-like proficiency compared to adult learners (Hartshorne et al., 2018, Newport, 1990). Several hypotheses have been proposed to explain this paradox in second language learning. One is the ‘Less is More” hypothesis, which claims that age-related limitations in cognitive capacity reduce the size of the processing window available for language learning, resulting in children focusing on smaller chunks of language, which facilitates local pattern learning (e.g., generalization in rule formation). This type of learning takes time in the beginning but is eventually more beneficial for ultimate attainment (Newport, 2020). Our fMRI results are broadly consistent with this hypothesis. Specifically, the limited activation of the working memory and syntax related brain areas in the younger participants aligns with the idea that they have not yet reached adult-like cognitive capacity (Simmonds et al., 2017). This cognitive constraint could narrow the processing window available to children when acquiring grammar, allowing them, but only at a later point in time, to master important local morphosyntactic dependencies (e.g., as in the morphological paradigms tested here) as a basis for the complete L2 grammar system. On this view, the end point of brain maturation in late adolescence has seemingly paradoxical effects. On the one hand, it means that reaching late adolescence is beneficial for relatively fast and successful (but not perfect) learning of a new morphosyntactic system, as we have shown here. On the other hand, it could bring an end to the long-term benefit on grammar learning of an earlier age of acquisition that in turn could ultimately contribute to higher attainment levels for younger learners. Although our findings do not speak directly to this issue, because we did not measure ultimate L2 proficiency, we note that they are consistent with the “Less is More” hypothesis.

Another frequently described neurobiological theory of language is the Declarative/Procedural model (Ullman, 2004), which proposes that children and adults rely on different memory systems. According to this model, children rely on their procedural memory abilities to learn a new grammar, since not all brain regions essential for declarative memory processes are fully developed. In contrast, adults tend to over-rely on their—fully matured—declarative memory abilities and use explicit learning strategies, especially during the initial learning phase (Hamrick et al., 2018, Morgan-Short et al., 2014). Recent neuroimaging studies substantiate this behavioral model by demonstrating that the dorsolateral prefrontal cortex (DLPFC) influences whether language learning relies on procedural or declarative memory processes (Ambrus et al., 2020): Experimentally, disruption of the DLPFC in healthy adults using transcranial magnetic stimulation promoted procedural learning. This is consistent with the idea in the procedural/declarative model that, because the DLPFC is still under development, young individuals must rely mainly on procedural learning processes. Our finding of continuity of activation in the brain networks (Fig. 3) despite discontinuity in the behavioral data (Fig. 2) appears to be inconsistent with the Declarative/Procedural model. We note, however, that the model’s two systems may be differentially implicated in other components of language learning and/or under different learning conditions.

Our study has several methodological strengths. First, we applied a whole-brain analysis approach within a large-scale continuous developmental sample; second, we developed a multi-level grammar learning paradigm which allowed us to investigate the direct effects of age without floor and/or ceiling effects across a broad age range during the initial phase of grammar learning. Moreover, even from a purely behavioral viewpoint, we are not aware of any grammar learning study on a participant sample with such a large continuous age range. A power analysis indicated that a developmental sample of 28 participants was required to detect the observed medium-to-large effect size (*f*^2^ =.25). This means our large sample size was sufficient to answer our main research questions. The sample lacked power to detect age-related differences within each individual age year; however, the literature has shown that age-related differences in cognitive functioning during development are often gradual and, therefore, not detectable in narrow age groups. Although our study is open to the criticism that we did not test a full linguistic system, we believe our language task was a key strength of our study, enabling us to control the amount of learning experience of Icelandic grammar for each participant while ensuring grammatical learning was not confounded by vocabulary learning. However, there are some limitations to our work. First, most of the older participants have learned multiple additional languages. In the Netherlands, pupils learn new languages already in the early high school years starting around the age of twelve, and English is taught (starting at different ages in different schools but no later than age 11) in primary school. Since we discovered a turning point around the age of 15.4, however, we do not think that this L2 instruction affected our results. Second, we cannot rule out the possibility that the observed age-related effects in brain activation may reflect, in part, differences in grammatical processing rather than learning. However, it is crucial to highlight that participants must have learned the grammar first in order to process and comprehend the new grammatical system. This implies that the effects observed are intricately linked to the acquisition of the morphosyntactic rules rather than solely due to processing. Thirdly, this study has focused on the initial learning phase using a cross-sectional design. For this reason, we are unable to examine brain activation and its relation to grammar learning performance longitudinally and/or after long-term learning.

## 5 Conclusion

This study investigated the neural correlates of age-related variability in initial grammar acquisition across a large developmental sample. Consistent with past studies (Snow and Hoefnagel-Hohle, 1978), we found age-related differences in grammar learning performance, in the initial learning phase, which has not previously been investigated in a large-scale developmental sample. Additionally, our data shows that the effect of age on grammar acquisition shifts at a similar temporal turning point, that is, late adolescence, as reported in a previous large-scale study on long-term L2 proficiency (Hartshorne et al., 2018). Furthermore, we are the first to link this age-related variability in grammar learning performance with neural activation: the older the individual, the more they activate networks related to working memory and language-specific processing. Altogether, this study suggests that the maturation of these specific networks could explain the observed age-related variability in (early phase) grammar acquisition. Moreover, our results indicate that children and adults rely on similar brain regions for grammar learning, and that activation within this network is age-dependent, rather than that they rely on different networks. This study provides links between age-related effects and neural underpinnings in relation to grammar learning, and thus lays the foundation for future research to further investigate how brain development can affect ultimate attainment in second language acquisition.

## Declaration of Competing Interest

The authors declare that they have no known competing financial interests or personal relationships that could have appeared to influence the work reported in this paper.

## Data Availability

Due to the young participants in our dataset, not all raw data could be shared. Data not available / The data that has been used is confidential.
