# Supplementary material for: Developmental changes in brain activation during novel grammar learning in 8-25-year-olds
Source: Dev Cogn Neurosci. 2024 Jan 20;66:101347. doi: 10.1016/j.dcn.2024.101347 (PMC10839867; doi:10.1016/j.dcn.2024.101347)
Supplement: Table S1 — Supplementary material [file mmc1.docx]

**----------- Supplement ---------------**


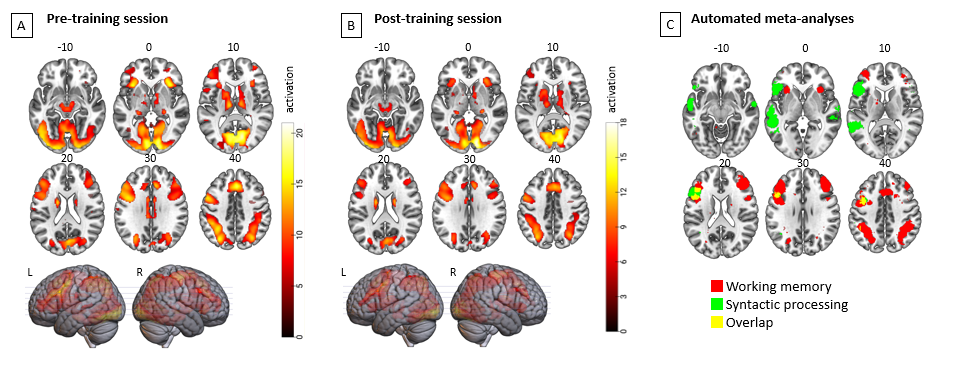


***Fig. S1.*** *Activation during grammaticality judgment task for the (A) pre- and (B) post-training session. Areas of increased activation are shown in warm (red-yellow) scale (cluster-level, p < .05 FWE-corrected). (C) Overview of the working memory related areas (red), overall activation during syntactic tasks (green), and their overlapping regions (yellow) based on automated meta-analyses from neurosynth.org (ref: doi:10.1038/nmeth.1635).*

***Table S1.*** *Peak activation of clusters (surviving FWE-correction p < .05; ≥30 voxels) for the whole sample during GJT for the pre- and post-training session.*

|  |  |  |  | local maxima | | |  |  |  |
| --- | --- | --- | --- | --- | --- | --- | --- | --- | --- |
| *#* | Region | L/R | vol | x | y | z | t | *p-value* |  |
| **pre-training** | |  |  |  |  |  |  |  |  |
| 1 | Cerebellum, vermis, calcarine gyrus | L/R | 42159 | -6 | -74 | -22 | 22.06 | .000 |  |
| 2 | SMA | L/R | 3298 | 0 | 16 | 48 | 21.92 | .000 |  |
| 3 | Middle/superior cingulate | L | 756 | -6 | -28 | 26 | 13.18 | .000 |  |
| 4 | Anterior/medial orbital gyrus | L | 87 | -18 | 46 | -14 | 11.40 | .000 |  |
| 5 | Middle temporal gyrus | L | 322 | -50 | -40 | 8 | 8.80 | .000 |  |
| 6 | Postcentral gyrus | L | 142 | -58 | -16 | 24 | 8.23 | .000 |  |
| 7 | Caudate nucleus | L | 79 | -6 | 22 | 6 | 6.40 | .000 |  |
| **post-training** | |  |  |  |  |  |  |  |  |
| 1 | Cerebellum, vermis, calcarine gyrus | L/R | 29868 | 14 | -84 | 0 | 21.73 | .000 |  |
| 2 | SMA, precentral gyrus | L/R | 6980 | 0 | 18 | 46 | 17.52 | .000 |  |
| 3 | Inferior frontal gyrus (triangular), precentral gyrus | R | 1222 | 44 | 30 | 28 | 10.89 | .000 |  |
| 4 | Hippocampus | L | 219 | -22 | -30 | -4 | 10.61 | .000 |  |
| 5 | Superior anterior cingulate, middle cingulate | L | 218 | -4 | 0 | 28 | 9.37 | .000 |  |
|  |  |  |  |  |  |  |  |  |  |
| *All ×, y, z-coordinates represent local maxima in MNI space. p < .050 FWE-corrected, k = voxel.* | | | | | | | | |  |
|  |  |  |  |  |  |  |  |  |  |
|  |  |  |  |  |  |  |  |  |  |

***
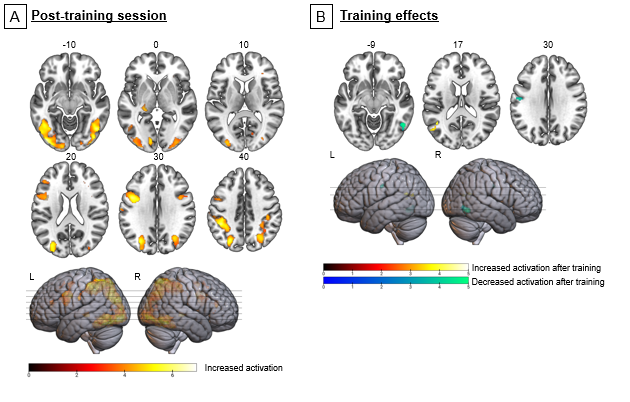
***

***Fig. S2.*** *Statistical parametric map (cluster-level, p < .05 FWE-corrected) displaying whole-brain increased (red-yellow) activation during grammatical processing in relation to the LOESS fitted curve, i.e., the combined effects of age(-related) and grammar learning performance during [A] the* *post-training session and [B] in relation to training effects.*

***Table S2.*** *Peak activation for clusters that showed whole-brain increased activation during grammatical processing in relation to the LOESS fitted curve, i.e., the combined effects of age(-related) and grammar learning performance during the pre-training session, post-training session, and in relation to training effects.*

|  |  |  |  | local maxima | | |  |  |  |
| --- | --- | --- | --- | --- | --- | --- | --- | --- | --- |
| *#* | Region | L/R | vol | x | y | z | t | *p-value* |  |
| **pre-training** | |  |  |  |  |  |  |  |  |
| 1 | Inferior temporal - fusiform gyrus | R | 4730 | 52 | -56 | -14 | 6.47 | .000 |  |
| 2 | Precentral, IFG (triangular) | L | 1079 | -42 | 6 | 32 | 6.04 | .000 |  |
| 3 | Inferior parietal gyrus | L | 2738 | -42 | -48 | 54 | 5.99 | .000 |  |
| 4 | Fusiform - inferior occipital gyrus | L | 1613 | -44 | -64 | -14 | 5.46 | .000 |  |
| 5 | Vermis | L | 167 | -2 | -60 | -26 | 4.61 | .008 |  |
| 6 | Precentral gyrus | R | 242 | 58 | 6 | 30 | 4.21 | .000 |  |
| **post-training** | |  |  |  |  |  |  |  |  |
| 1 | Inferior parietal gyrus, fusiform gyrus | L | 7124 | -42 | -42 | 44 | 7.13 | .000 |  |
| 2 | Precentral, IFG (opercular) | L | 1208 | -44 | 6 | 32 | 6.73 | .000 |  |
| 3 | Inferior temporal gyrus, superior/inferior parietal gyrus | R | 4842 | 48 | -64 | -12 | 6.52 | .000 |  |
| 4 | Thalamus | L | 165 | -20 | -26 | -4 | 5.99 | .006 |  |
| 5 | Precentral gyrus | R | 216 | 40 | 4 | 28 | 4.73 | .001 |  |
| 6 | IFG (triangular), middle frontal gyrus | R | 156 | 32 | 32 | 12 | 4.65 | .009 |  |
| 7 | Superior frontal gyrus | L | 160 | -20 | 0 | 50 | 4.58 | .008 |  |
| 8 | IFG (triangular) | L | 154 | -44 | 34 | 20 | 4.28 | .009 |  |
| 9 | SMA, superior frontal gyrus | R | 175 | 6 | 18 | 46 | 4.07 | .004 |  |
| 10 | Calcarine gyrus | R | 107 | 12 | -72 | 14 | 3.76 | .048 |  |
| **training effects** | |  |  |  |  |  |  |  |  |
| *increased activation* | |  |  |  |  |  |  |  |  |
| 1 | Middle temporal gyrus | L | 143 | -42 | -56 | 18 | 4.39 | .012 |  |
| *decreased activation* | |  |  |  |  |  |  |  |  |
| 1 | Inferior/middle temporal gyrus | R | 272 | 52 | -58 | -10 | 5.02 | .000 |  |
| 2 | Postcentral gyrus | L | 109 | -52 | -14 | 32 | 4.63 | .042 |  |
| *All ×, y, z-coordinates represent local maxima in MNI space. Cluster threshold p < .001; p < .050 FWE-corrected, k = voxel. IFG = inferior frontal gyrus* | | | | | | | | |  |
|  |  |  |  |  |  |  |  |  |  |
|  |  |  |  |  |  |  |  |  |  |


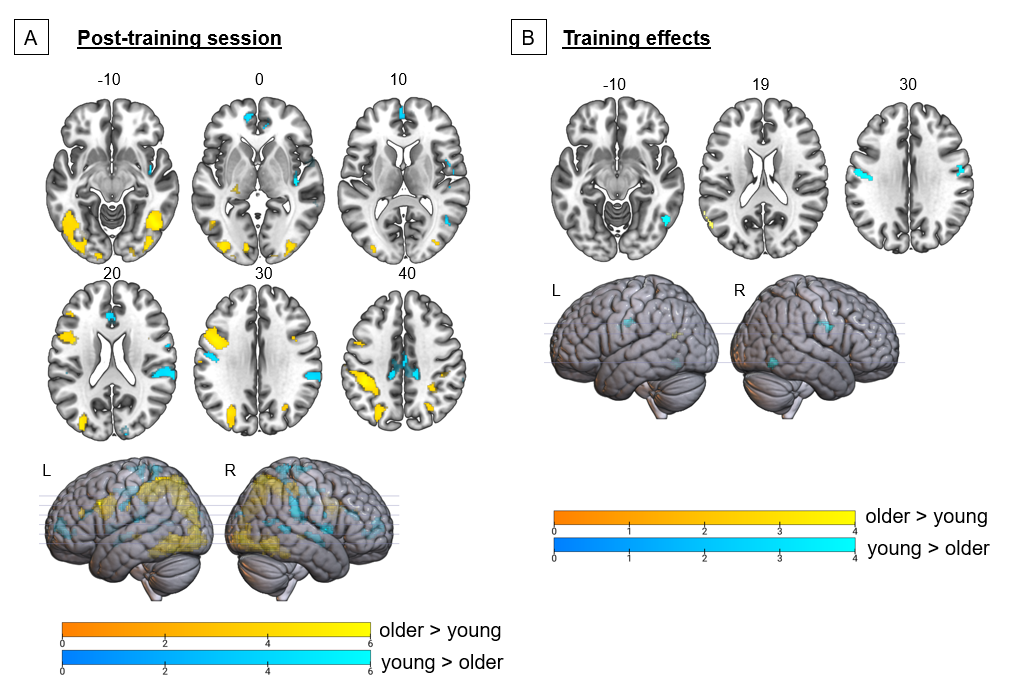
***Fig. S3.*** *Group comparison of the older group (15.4-25 years) to the young group (8 until 15.4 years) displaying whole-brain increased (yellow) and reduced (blue) activation for the older group compared to the young group during grammatical processing at [A] the* *post-training session and [B] in relation to training effects. Cluster-level (p <.001) corrected, p <.05 FWE-corrected; MNI space)*

***Table S3.*** *Peak activation for clusters that differed between the older group and the young group during grammatical processing during the pre-training session, post-training session, and in relation to training effects.*

|  |  |  |  | local maxima | | |  |  |  |
| --- | --- | --- | --- | --- | --- | --- | --- | --- | --- |
| *#* | Region | L/R | vol | x | y | z | t | *p-value* |  |
| **pre-training** | |  |  |  |  |  |  |  |  |
| *young>older* | |  |  |  |  |  |  |  |  |
| 1 | Superior ACC | R | 2512 | 2 | 22 | 20 | 6.32 | .000 |  |
| 2 | Middle cingulate | L | 1931 | -8 | -30 | 40 | 6.44 | .000 |  |
| 3 | Superior temporal/Supramarginal gyrus | R | 891 | 50 | -32 | 22 | 5.21 | .000 |  |
| 4 | Middle temporal/inferior parietal gyrus | L | 524 | -52 | -66 | 20 | 5.04 | .000 |  |
| 5 | Insula | R | 173 | 40 | -4 | -8 | 4.97 | .006 |  |
| 6 | Superior temporal/ Supramarginal gyrus | L | 166 | -56 | -24 | 12 | 4.61 | .008 |  |
| 7 | Middle/Superior frontal gyrus | R | 165 | 16 | 50 | 38 | 4.80 | .008 |  |
| 8 | Superior temporal gyrus | L | 149 | -40 | -14 | -6 | 5.37 | .014 |  |
| 9 | Hippocampus | R | 121 | 32 | -32 | -8 | 4.53 | .035 |  |
| 10 | Insula | L | 117 | -36 | 14 | -10 | 5.66 | .040 |  |
| *older>young* | |  |  |  |  |  |  |  |  |
| 1 | Inferior/superior parietal gyrus | L | 2541 | -28 | -70 | 52 | 5.94 | .000 |  |
| 2 | Inferior/superior parietal gyrus | R | 2260 | 34 | -64 | 56 | 5.76 | .000 |  |
| 3 | Inferior occipital gyrus | R | 1697 | 44 | -62 | -16 | 7.16 | .000 |  |
| 4 | Fusiform gyrus, inferior occipital gyrus | L | 1439 | -44 | -62 | -16 | 5.35 | .000 |  |
| 5 | IFG (opercular/triangular), precentral | L | 1221 | -52 | 14 | 32 | 6.48 | .000 |  |
| 6 | IFG (opercular/triangular), precentral | R | 547 | 54 | 12 | 32 | 4.94 | .000 |  |
| 7 | Caudate nucleus | L/R | 328 | 8 | 8 | 14 | 6.10 | .000 |  |
| 8 | SMA, superior frontal gyrus | L | 186 | -4 | 12 | 52 | 4.51 | .004 |  |
| 9 | Vermis | R | 174 | 2 | -54 | -22 | 4.52 | .006 |  |
| 10 | Insula | R | 133 | 38 | 28 | 0 | 4.55 | .024 |  |
| **post-training** | |  |  |  |  |  |  |  |  |
| *young>older* | |  |  |  |  |  |  |  |  |
| 1 | Middle cingulate, precentral | R | 2089 | 2 | -18 | 46 | 5.71 | .000 |  |
| 2 | Superior temporal/Supramarginal gyrus | R | 1161 | 64 | -32 | 22 | 5.37 | .000 |  |
| 3 | Pregenual ACC, Superior frontal gyrus (medial) | L | 574 | -10 | 50 | 0 | 4.51 | .000 |  |
| 4 | Superior ACC | L | 219 | -4 | 32 | 20 | 5.18 | .001 |  |
| 5 | Postcentral gyrus | L | 198 | -46 | -14 | 30 | 4.75 | .002 |  |
| 6 | Insula | R | 197 | 40 | -18 | 0 | 5.51 | .002 |  |
| 7 | Postcentral gyrus, paracentral Lobule | L | 170 | -20 | -28 | 64 | 4.33 | .005 |  |
| 8 | Middle temporal gyrus | R | 149 | 64 | -48 | 8 | 4.67 | .011 |  |
| 9 | Middle temporal gyrus | R | 148 | 46 | -60 | 10 | 4.34 | .012 |  |
| 10 | Superior temporal gyrus | L | 131 | -46 | -30 | 16 | 4.28 | .021 |  |
| 11 | Superior occipital gyrus, cuneus | R | 121 | 14 | -96 | 18 | 4.14 | .030 |  |
| *older>young* | |  |  |  |  |  |  |  |  |
| 1 | Inferior/superior parietal gyrus | L | 5892 | -42 | -40 | 44 | 6.54 | .000 |  |
| 2 | Inferior temporal/occipital gyrus | R | 1737 | 46 | -62 | -12 | 6.71 | .000 |  |
| 3 | Superior/inferior parietal gyrus | R | 1656 | 26 | -68 | 58 | 5.46 | .000 |  |
| 4 | Precentral gyrus | L | 1170 | -44 | 6 | 32 | 6.08 | .000 |  |
| 5 | Precentral gyrus | R | 124 | 42 | 4 | 28 | 4.47 | .027 |  |
| 6 | Hippocampus, thalamus | L | 116 | -20 | -24 | -4 | 4.76 | .035 |  |
| 7 | IFG (triangular) | L | 114 | -46 | 34 | 22 | 4.04 | .038 |  |
| **training effects** | |  |  |  |  |  |  |  |  |
| *young>older* | |  |  |  |  |  |  |  |  |
| 1 | Postcentral/precentral gyrus, IFG (opercular) | R | 232 | 52 | -12 | 30 | 4.06 | .000 |  |
| 2 | Postcentral/precentral gyrus | L | 206 | -52 | -14 | 32 | 4.29 | .001 |  |
| 3 | Inferior temporal/occipital gyrus | R | 166 | 52 | -60 | -12 | 4.84 | .005 |  |
| *older>young* | |  |  |  |  |  |  |  |  |
| - |  |  |  |  |  |  |  |  |  |
| *All ×, y, z-coordinates represent local maxima in MNI space. Cluster threshold p < .001; p < .050 FWE-corrected, k = voxel. IFG = inferior frontal gyrus* | | | | | | | | |  |
|  |  |  |  |  |  |  |  |  |  |

***Table S4.*** *Peak activation cluster report for each group (young group and older group) in relation to grammar learning performance, independent of age.*

|  |  |  |  | local maxima | | |  |  |
| --- | --- | --- | --- | --- | --- | --- | --- | --- |
| *#* | Region | L/R | vol | x | y | z | t | *p-value* |
| **pre-training** | |  |  |  |  |  |  |  |
| *young group* | |  |  |  |  |  |  |  |
| 1 | Cerebellum, calcarine gyrus | L | 1908 | -8 | -72 | -22 | 7.08 | .000 |
| 2 | Superior parietal/occipital gyrus | R | 688 | 22 | -52 | 56 | 5.28 | .000 |
| 3 | SMA | R | 454 | 8 | 14 | 52 | 5.76 | .000 |
| 4 | Inferior temporal gyrus | L | 421 | -50 | -62 | -10 | 6.15 | .000 |
| 5 | Inferior/superior parietal gyrus | L | 373 | -34 | -54 | 48 | 5.85 | .000 |
| 6 | Precentral gyrus, IFG (opercular) | L | 308 | -50 | 8 | 32 | 5.12 | .000 |
| 7 | Cerebellum | R | 268 | 26 | -62 | -28 | 5.30 | .000 |
| 8 | Cerebellum | L | 229 | -32 | -66 | -26 | 6.42 | .000 |
| 9 | Inferior parietal gyrus | L | 183 | -48 | -40 | 42 | 5.72 | .003 |
| 10 | Cuneus, precuneus gyrus | R | 162 | 16 | -68 | 30 | 4.95 | .006 |
| 11 | Middle/superior occipital gyrus | R | 140 | 34 | -76 | 30 | 5.28 | .014 |
| 12 | Precentral gyrus, middle frontal gyrus | R | 138 | 36 | -6 | 56 | 5.43 | .015 |
| 13 | Precentral gyrus | L | 114 | -42 | -8 | 60 | 3.98 | .035 |
| 14 | Postcentral/precentral gyrus | R | 108 | 44 | -30 | 46 | 4.87 | .044 |
| *older group* | |  |  |  |  |  |  |  |
| 1 | Middle occipital gyrus, angular gyrus | R | 2050 | 32 | -72 | 32 | 5.16 | .000 |
| 2 | Superior/inferior parietal gyrus | L | 620 | -34 | -64 | 54 | 5.99 | .000 |
| 3 | Precentral, IFG (opercular) | L | 425 | -44 | 8 | 32 | 4.92 | .000 |
| 4 | Fusiform gyrus, cerebellum | L | 415 | -34 | -66 | -18 | 4.79 | .000 |
| 5 | IFG (triangular/opercular) | R | 253 | 32 | 22 | 28 | 4.41 | .000 |
| 6 | Inferior temporal gyrus | L | 154 | -54 | -56 | -20 | 4.33 | .007 |
| 7 | Superior/middle frontal gyrus | R | 148 | 28 | 2 | 48 | 5.15 | .009 |
| 8 | Middle/superior frontal gyrus | L | 148 | -28 | 8 | 60 | 4.28 | .009 |
| 9 | Inferior parietal gyrus | L | 137 | -46 | -40 | 42 | 5.54 | .013 |
| **post-training** | |  |  |  |  |  |  |  |
| *young group* | |  |  |  |  |  |  |  |
| 1 | Calcarine gyrus | L/R | 468 | 4 | -84 | 2 | 6.02 | .000 |
| 2 | Superior parietal gyrus | R | 221 | 26 | -52 | 56 | 5.33 | .001 |
| *older group* | |  |  |  |  |  |  |  |
| 1 | Lingual gyrus | R | 135 | 16 | -74 | -10 | 4.41 | .014 |
|  |  |  |  |  |  |  |  |  |
| *All ×, y, z-coordinates represent local maxima in MNI space. Cluster threshold p < .001; p < .050 FWE-corrected, k = voxel. IFG = inferior frontal gyrus* | | | | | | | | |
|  |  |  |  |  |  |  |  |  |
|  |  |  |  |  |  |  |  |  |

**Supplement: L1 grammar proficiency**

We measured the participants’ L1 Dutch grammatical proficiency, using the Syntest, as part of the larger study (Menks et al., 2022). Although first language grammar proficiency was positively correlated with GJT performance (R^2^=.13, p < .001) and age (R^2^=.08, p < .001), the fMRI results did not significantly differ when adding L1 grammar proficiency as a covariate next to the age-related differences in L2 grammar score (see Fig. S4). Additionally, we observed no cluster that correlated with L1 grammar proficiency when controlling for L2 grammar performance, indicating that L1 grammar proficiency had no significant effect on the fMRI results.”


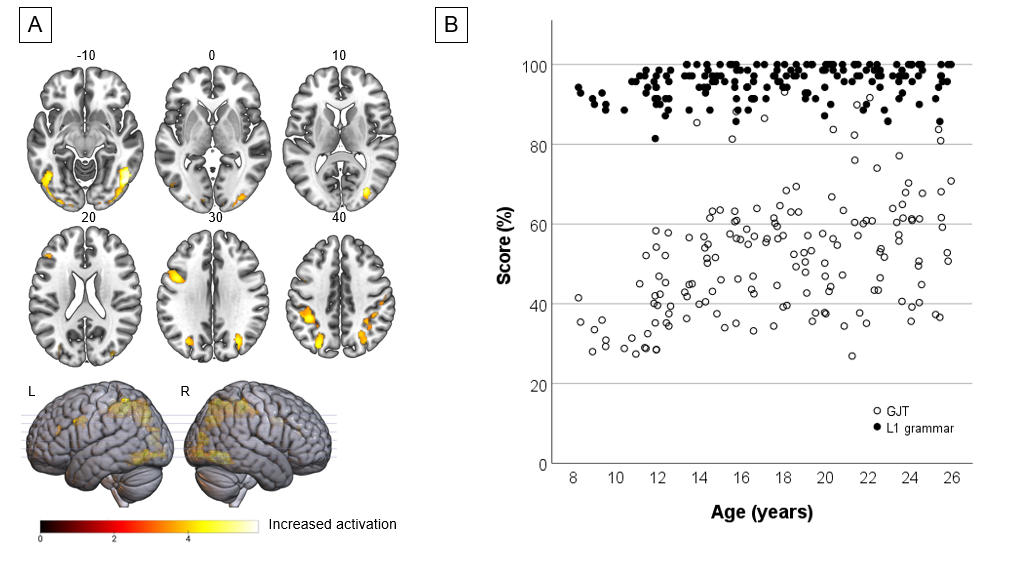


*Fig. S4. [A] Statistical parametric map (cluster-level, p < .05 FWE-corrected) displaying whole-brain increased (red-yellow) activation during grammatical processing in relation to the LOESS fitted curve, controlled for first language (L1) grammar proficiency. [B] Age-related effect in the grammaticality judgment task (GJT) and the L1 language grammar test scores (Syntest; Dutch) of the participants.*

**Supplement: Preprocessing pipeline (fMRIprep)**

Results included in this manuscript are preprocessed using fMRIPrep 22.0.1

**Preprocessing of B0 inhomogeneity mappings**

A total of two fieldmaps were found available within the input BIDS structure for this particular subject. A B0 nonuniformity map (or fieldmap) was estimated from the phase-drift map(s) measure with two consecutive GRE (gradient-recalled echo) acquisitions. The corresponding phase-map(s) were phase-unwrapped with prelude (FSL 6.0.5.1:57b01774) and then skull-stripped. Due to mechanical issues, the fieldmap of two participants were not collected during the second fMRI session, and thus no fieldmap correction was applied to their second session dataset. Analyses showed no significant alteration in the overall group effect for including these participants, therefore we have included both participants in our study.

**Anatomical data preprocessing**

A total of 2 T1-weighted (T1w) images were found within the input BIDS dataset. All of them were corrected for intensity non-uniformity (INU) with N4BiasFieldCorrection (Tustison et al. 2010), distributed with ANTs 2.3.3 (Avants et al. 2008, RRID:SCR_004757). The T1w-reference was then skull-stripped with a Nipype implementation of the antsBrainExtraction.sh workflow (from ANTs), using OASIS30ANTs as target template. Brain tissue segmentation of cerebrospinal fluid (CSF), white-matter (WM) and gray-matter (GM) was performed on the brain-extracted T1w using fast (FSL 6.0.5.1:57b01774, RRID:SCR_002823, Zhang, Brady, and Smith 2001). A T1w-reference map was computed after registration of 2 T1w images (after INU-correction) using mri_robust_template (FreeSurfer 7.2.0, Reuter, Rosas, and Fischl 2010). Brain surfaces were reconstructed using recon-all (FreeSurfer 7.2.0, RRID:SCR_001847, Dale, Fischl, and Sereno 1999), and the brain mask estimated previously was refined with a custom variation of the method to reconcile ANTs-derived and FreeSurfer-derived segmentations of the cortical gray-matter of Mindboggle (RRID:SCR_002438, Klein et al. 2017). Volume-based spatial normalization to two standard spaces (MNI152NLin6Asym, MNI152NLin2009cAsym) was performed through nonlinear registration with antsRegistration (ANTs 2.3.3), using brain-extracted versions of both T1w reference and the T1w template. The following templates were selected for spatial normalization: FSL’s MNI ICBM 152 non-linear 6th Generation Asymmetric Average Brain Stereotaxic Registration Model [Evans et al. (2012), RRID:SCR_002823; TemplateFlow ID: MNI152NLin6Asym], ICBM 152 Nonlinear Asymmetrical template version 2009c [Fonov et al. (2009), RRID:SCR_008796; TemplateFlow ID: MNI152NLin2009cAsym].

**Functional data preprocessing**

For each of BOLD run found per subject (across all tasks and sessions), the following preprocessing was performed. First, a reference volume and its skull-stripped version were generated by aligning and averaging 3 single-band references (SBRefs). Head-motion parameters with respect to the BOLD reference (transformation matrices, and six corresponding rotation and translation parameters) are estimated before any spatiotemporal filtering using mcflirt (FSL 6.0.5.1:57b01774, Jenkinson et al. 2002). The estimated skull-stripped fieldmap was then aligned with rigid-registration to the target EPI (echo-planar imaging) reference run. The field coefficients were mapped on to the reference EPI using the transform. A T2★ map was estimated from the preprocessed EPI echoes, by voxel-wise fitting the maximal number of echoes with reliable signal in that voxel to a monoexponential signal decay model with nonlinear regression. The T2★/S0 estimates from a log-linear regression fit were used for initial values. The calculated T2★ map was then used to optimally combine preprocessed BOLD across echoes following the method described in (Posse et al. 1999). The optimally combined time series was carried forward as the preprocessed BOLD. The BOLD reference was then co-registered to the T1w reference using bbregister (FreeSurfer) which implements boundary-based registration (Greve and Fischl 2009). Co-registration was configured with six degrees of freedom. First, a reference volume and its skull-stripped version were generated using a custom methodology of fMRIPrep. Several confounding time-series were calculated based on the preprocessed BOLD: framewise displacement (FD), DVARS and three region-wise global signals. FD was computed using two formulations following Power (absolute sum of relative motions, Power et al. (2014)) and Jenkinson (relative root mean square displacement between affines, Jenkinson et al. (2002)). FD and DVARS are calculated for each functional run, both using their implementations in Nipype (following the definitions by Power et al. 2014). The three global signals are extracted within the CSF, the WM, and the whole-brain masks. Additionally, a set of physiological regressors were extracted to allow for component-based noise correction (CompCor, Behzadi et al. 2007). Principal components are estimated after high-pass filtering the preprocessed BOLD time-series (using a discrete cosine filter with 128s cut-off) for the two CompCor variants: temporal (tCompCor) and anatomical (aCompCor). tCompCor components are then calculated from the top 2% variable voxels within the brain mask. For aCompCor, three probabilistic masks (CSF, WM and combined CSF+WM) are generated in anatomical space. The implementation differs from that of Behzadi et al. in that instead of eroding the masks by 2 pixels on BOLD space, a mask of pixels that likely contain a volume fraction of GM is subtracted from the aCompCor masks. This mask is obtained by dilating a GM mask extracted from the FreeSurfer’s aseg segmentation, and it ensures components are not extracted from voxels containing a minimal fraction of GM. Finally, these masks are resampled into BOLD space and binarized by thresholding at 0.99 (as in the original implementation). Components are also calculated separately within the WM and CSF masks. For each CompCor decomposition, the k components with the largest singular values are retained, such that the retained components’ time series are sufficient to explain 50 percent of variance across the nuisance mask (CSF, WM, combined, or temporal). The remaining components are dropped from consideration. The head-motion estimates calculated in the correction step were also placed within the corresponding confounds file. The confound time series derived from head motion estimates and global signals were expanded with the inclusion of temporal derivatives and quadratic terms for each (Satterthwaite et al. 2013). Frames that exceeded a threshold of -0.6 mm FD or 1.5 standardized DVARS were annotated as motion outliers. Additional nuisance timeseries are calculated by means of principal components analysis of the signal found within a thin band (crown) of voxels around the edge of the brain, as proposed by (Patriat, Reynolds, and Birn 2017). The BOLD time-series were resampled into standard space, generating a preprocessed BOLD run in MNI152NLin6Asym space. First, a reference volume and its skull-stripped version were generated using a custom methodology of fMRIPrep. Automatic removal of motion artifacts using independent component analysis (ICA-AROMA, Pruim et al. 2015) was performed on the preprocessed BOLD on MNI space time-series after removal of non-steady state volumes and spatial smoothing with an isotropic, Gaussian kernel of 6mm FWHM (full-width half-maximum). Corresponding “non-aggresively” denoised runs were produced after such smoothing. Additionally, the “aggressive” noise-regressors were collected and placed in the corresponding confounds file. All resamplings can be performed with a single interpolation step by composing all the pertinent transformations (i.e. head-motion transform matrices, susceptibility distortion correction when available, and co-registrations to anatomical and output spaces). Gridded (volumetric) resamplings were performed using antsApplyTransforms (ANTs), configured with Lanczos interpolation to minimize the smoothing effects of other kernels (Lanczos 1964). Non-gridded (surface) resamplings were performed using mri_vol2surf (FreeSurfer).

**References fMRI prep**

*Abraham, Alexandre, Fabian Pedregosa, Michael Eickenberg, Philippe Gervais, Andreas Mueller, Jean Kossaifi, Alexandre Gramfort, Bertrand Thirion, and Gael Varoquaux. 2014. “Machine Learning for Neuroimaging with Scikit-Learn.” Frontiers in Neuroinformatics 8. https://doi.org/10.3389/fninf.2014.00014.*

*Avants, B. B., C. L. Epstein, M. Grossman, and J. C. Gee. 2008. “Symmetric Diffeomorphic Image Registration with Cross-Correlation: Evaluating Automated Labeling of Elderly and Neurodegenerative Brain.” Medical Image Analysis 12 (1): 26–41. https://doi.org/10.1016/j.media.2007.06.004.*

*Behzadi, Yashar, Khaled Restom, Joy Liau, and Thomas T. Liu. 2007. “A Component Based Noise Correction Method (CompCor) for BOLD and Perfusion Based fMRI.” NeuroImage 37 (1): 90–101. https://doi.org/10.1016/j.neuroimage.2007.04.042.*

*Dale, Anders M., Bruce Fischl, and Martin I. Sereno. 1999. “Cortical Surface-Based Analysis: I. Segmentation and Surface Reconstruction.” NeuroImage 9 (2): 179–94. https://doi.org/10.1006/nimg.1998.0395.*

*Esteban, Oscar, Ross Blair, Christopher J. Markiewicz, Shoshana L. Berleant, Craig Moodie, Feilong Ma, Ayse Ilkay Isik, et al. 2018. “fMRIPrep 22.0.1.” Software. https://doi.org/10.5281/zenodo.852659.*

*Esteban, Oscar, Christopher Markiewicz, Ross W Blair, Craig Moodie, Ayse Ilkay Isik, Asier Erramuzpe Aliaga, James Kent, et al. 2018. “fMRIPrep: A Robust Preprocessing Pipeline for Functional MRI.” Nature Methods. https://doi.org/10.1038/s41592-018-0235-4.*

*Evans, AC, AL Janke, DL Collins, and S Baillet. 2012. “Brain Templates and Atlases.” NeuroImage 62 (2): 911–22. https://doi.org/10.1016/j.neuroimage.2012.01.024.*

*Fonov, VS, AC Evans, RC McKinstry, CR Almli, and DL Collins. 2009. “Unbiased Nonlinear Average Age-Appropriate Brain Templates from Birth to Adulthood.” NeuroImage 47, Supplement 1: S102. https://doi.org/10.1016/S1053-8119(09)70884-5.*

*Gorgolewski, K., C. D. Burns, C. Madison, D. Clark, Y. O. Halchenko, M. L. Waskom, and S. Ghosh. 2011. “Nipype: A Flexible, Lightweight and Extensible Neuroimaging Data Processing Framework in Python.” Frontiers in Neuroinformatics 5: 13. https://doi.org/10.3389/fninf.2011.00013.*

*Gorgolewski, Krzysztof J., Oscar Esteban, Christopher J. Markiewicz, Erik Ziegler, David Gage Ellis, Michael Philipp Notter, Dorota Jarecka, et al. 2018. “Nipype.” Software. https://doi.org/10.5281/zenodo.596855.*

*Greve, Douglas N, and Bruce Fischl. 2009. “Accurate and Robust Brain Image Alignment Using Boundary-Based Registration.” NeuroImage 48 (1): 63–72. https://doi.org/10.1016/j.neuroimage.2009.06.060.*

*Jenkinson, Mark, Peter Bannister, Michael Brady, and Stephen Smith. 2002. “Improved Optimization for the Robust and Accurate Linear Registration and Motion Correction of Brain Images.” NeuroImage 17 (2): 825–41. https://doi.org/10.1006/nimg.2002.1132.*

*Klein, Arno, Satrajit S. Ghosh, Forrest S. Bao, Joachim Giard, Yrjö Häme, Eliezer Stavsky, Noah Lee, et al. 2017. “Mindboggling Morphometry of Human Brains.” PLOS Computational Biology 13 (2): e1005350. https://doi.org/10.1371/journal.pcbi.1005350.*

*Lanczos, C. 1964. “Evaluation of Noisy Data.” Journal of the Society for Industrial and Applied Mathematics Series B Numerical Analysis 1 (1): 76–85. https://doi.org/10.1137/0701007.*

*Patriat, Rémi, Richard C. Reynolds, and Rasmus M. Birn. 2017. “An Improved Model of Motion-Related Signal Changes in fMRI.” NeuroImage 144, Part A (January): 74–82. https://doi.org/10.1016/j.neuroimage.2016.08.051.*

*Posse, Stefan, Stefan Wiese, Daniel Gembris, Klaus Mathiak, Christoph Kessler, Maria-Liisa Grosse-Ruyken, Barbara Elghahwagi, Todd Richards, Stephen R. Dager, and Valerij G. Kiselev. 1999. “Enhancement of BOLD-Contrast Sensitivity by Single-Shot Multi-Echo Functional MR Imaging.” Magnetic Resonance in Medicine 42 (1): 87–97. https://doi.org/10.1002/(SICI)1522-2594(199907)42:1<87::AID-MRM13>3.0.CO;2-O.*

*Power, Jonathan D., Anish Mitra, Timothy O. Laumann, Abraham Z. Snyder, Bradley L. Schlaggar, and Steven E. Petersen. 2014. “Methods to Detect, Characterize, and Remove Motion Artifact in Resting State fMRI.” NeuroImage 84 (Supplement C): 320–41. https://doi.org/10.1016/j.neuroimage.2013.08.048.*

*Pruim, Raimon H. R., Maarten Mennes, Daan van Rooij, Alberto Llera, Jan K. Buitelaar, and Christian F. Beckmann. 2015. “ICA-AROMA: A Robust ICA-Based Strategy for Removing Motion Artifacts from fMRI Data.” NeuroImage 112 (Supplement C): 267–77. https://doi.org/10.1016/j.neuroimage.2015.02.064.*

*Reuter, Martin, Herminia Diana Rosas, and Bruce Fischl. 2010. “Highly Accurate Inverse Consistent Registration: A Robust Approach.” NeuroImage 53 (4): 1181–96. https://doi.org/10.1016/j.neuroimage.2010.07.020.*

*Satterthwaite, Theodore D., Mark A. Elliott, Raphael T. Gerraty, Kosha Ruparel, James Loughead, Monica E. Calkins, Simon B. Eickhoff, et al. 2013. “An improved framework for confound regression and filtering for control of motion artifact in the preprocessing of resting-state functional connectivity data.” NeuroImage 64 (1): 240–56. https://doi.org/10.1016/j.neuroimage.2012.08.052.*

*Tustison, N. J., B. B. Avants, P. A. Cook, Y. Zheng, A. Egan, P. A. Yushkevich, and J. C. Gee. 2010. “N4itk: Improved N3 Bias Correction.” IEEE Transactions on Medical Imaging 29 (6): 1310–20. https://doi.org/10.1109/TMI.2010.2046908.*

*Zhang, Y., M. Brady, and S. Smith. 2001. “Segmentation of Brain MR Images Through a Hidden Markov Random Field Model and the Expectation-Maximization Algorithm.” IEEE Transactions on Medical Imaging 20 (1): 45–57. https://doi.org/10.1109/42.906424.*
